# 

**Published:** 1879-03-20

**Authors:** 


### *GIVE US CREDIT.*

A "*Western*" journal—yea, two journals out that way—copied from our pages without giving us credit therefor. Brethren, it was perhaps accidental, and the articles used of but little value; and it may be that the best of us make these oversights; in "*Brief*," however, we would say, let us not forget these little courtesies.

☞SUBSCRIBERS in arrears are requested to remit their subscriptions at once.

### *BUFFALO LITHIA WATER.*

See advertisement of this celebrated water, in our present issue.

